# Lymph Node Metastasis of Gastric Cancer

**DOI:** 10.3390/cancers3022141

**Published:** 2011-04-26

**Authors:** Tomonori Akagi, Norio Shiraishi, Seigo Kitano

**Affiliations:** 1 Oita University Faculty of Medicine, Department of Gastroenterological Surgery, 1-1 Idaigaoka, Hasama-machi, Oita 879-5593, Japan; E-Mail: geka1@oita-u.ac.jp; 2 Surgical division, Center for community medicine, Oita University, 1-1 Idaigaoka, Hasama-machi, Oita 879-5593, Japan; E-Mail: norioh@oita-u.ac.jp

**Keywords:** gastric cancer, lymph node metastasis, lymph node dissection

## Abstract

Despite a decrease in incidence in recent decades, gastric cancer is still one of the most common causes of cancer death worldwide [1]. In areas without screening for gastric cancer, it is diagnosed late and has a high frequency of nodal involvement [1]. Even in early gastric cancer (EGC), the incidence of lymph node (LN) metastasis exceeds 10%; it was reported to be 14.1% overall and was 4.8 to 23.6% depending on cancer depth [2]. It is important to evaluate LN status preoperatively for proper treatment strategy; however, sufficient results are not being obtained using various modalities. Surgery is the only effective intervention for cure or long-term survival. It is possible to cure local disease without distant metastasis by gastrectomy and LN dissection. However, there is no survival benefit from surgery for systemic disease with distant metastasis such as para-aortic lymph node metastasis [3]. Therefore, whether the disease is local or systemic is an important prognostic indicator for gastric cancer, and the debate continues over the importance of extended lymphadenectomy for gastric cancer. The concept of micro-metastasis has been described as a prognostic factor [4-9], and the biological mechanisms of LN metastasis are currently under study [10-12]. In this article, we review the status of LN metastasis including its molecular mechanisms and evaluate LN dissection for the treatment of gastric cancer.

## 1. The Incidence of Lymph Node (LN) Metastasis in Gastric Cancer

### 1.1. Early Gastric Cancer (EGC)

As proposed by the Japanese Society of Gastroenterological Endoscopy in 1962, EGC is defined as adenocarcinoma that is limited to the gastric mucosa or submucosa regardless of the involvement of regional lymph nodes (T1) [13]. Many studies have clarified the status of LN metastasis in EGC. The overall incidence of LN metastases in T1 EGC is 10 to 20% [2,14-17]. The characteristics of the tumor such as the size, cancer depth, histologic type, and the presence of lymphovascular invasion are important determinants of the likelihood of spread [2,18-20]. For example, the Roviello *et al.* study evaluating 652 cases of resected EGC [2] showed that the incidence of LN metastasis to be 14.1% overall: 4.8% *versus* 23.6% for mucosal *versus* submucosal cancer. Smaller cancers were significantly less likely to be associated with positive nodes: 9% *versus* 20% and 30% for tumors <2 cm, 2 to 4 cm, and >4 cm in diameter, respectively. In the Sano *et al.* study, well-differentiated type I and IIa T1 tumors of less than 2 cm in diameter, and nonulcerative type IIc T1 tumors of less than 1 cm in diameter, were associated with a low risk of LN metastases (1.7%) [19]. Such a volume of sufficient data has contributed to the development of indications for endoscopic treatment.

### 1.2. Advanced Gastric Cancer

The number of studies reviewing both the progression of LN metastasis from EGC to advanced gastric cancer (AGC) and the status of AGC is insufficient. A report from Japan suggested that >60% of untreated EGCs will progress to AGC within five years [21]. Nakajima *et al.* reported that the incidence of LN metastasis of gastric cancer with invasion to MP, SS, SE, SI were 52.2%, 66.9%, 74.4%, and 82.6%, respectively [22]. However, it is difficult to judge the presence and the extent of LN metastasis in AGC before operation. Two issues are related to evaluation of the incidence of LN metastasis in AGC: First, many factors, such as location, depth, size, macroscopic type, and histological type of the AGC, affect the incidence and distribution of LN metastasis. Second, the diagnosis of LN metastasis with resected specimens is affected by examination methods such as H and E staining, immunohistochemical staining, and reverse polymerase chain reaction. To predict the incidence and distribution of LN metastasis in detail before operation for AGC, a special modality such as the computer information system developed by Maruyama *et al.* is necessary [23].

## 2. Diagnosis of LN Metastasis in Gastric Cancer

The accuracy rate of imaging examinations of LN metastasis in gastric cancer is not high. Therefore, the purpose of the preoperative evaluation is to initially stratify patients into two clinical groups: those with locoregional (stage I to III) disease and those with systemic (stage IV) involvement. As preoperative examinations, endoscopy and barium meal examinations are routinely used to evaluate the cancerous lesion in the stomach. Abdominal ultrasound (US) examination and computed tomography (CT) are usually used to examine the presence of invasion to other organs and metastatic lesions, but their diagnostic accuracy is limited.

### 2.1. Abdominal US

There are few reports about the accuracy of preoperative LN status using abdominal ultrasonography. Isozaki *et al.* reported that the detection rate of LN metastasis by transabdominal US was 5% [24]. Due to problems with intraluminal gas, abdominal US of the gastrointestinal tract is not commonly used and has not been developed. Rather than abdominal US, a number of studies of the effectiveness of endoscopic ultrasonography (EUS) have been reported (described in section 2.3).

### 2.2. Abdominal CT

Dynamic CT scanning is usually performed early in the preoperative evaluation after a diagnosis of gastric cancer is made. CT is widely available and noninvasive. It is good for widely evaluating metastatic disease, especially hepatic metastases, ascites, and distant nodal spread. In 20 to 30% of patients with a negative CT, however, intraperitoneal disease will be found at either staging laparoscopy or at open exploration [25-27].

Another limitation of CT is its inability to accurately assess the depth of primary tumor invasion and the presence of LN involvement. CT accurately assesses the T stage of the primary tumor in only about 50 to 70% of cases [28-34]. The classification of nodal status is usually based on LN size, and sensitivity of CT for detecting regional nodal metastases is limited for involved nodes that are smaller than 0.8 cm [28,33]. Furthermore, false-positive findings may be attributed to inflammatory lymphadenopathy. In several series of patients undergoing staging CT for gastric cancer or gastric plus esophageal cancer, sensitivity and specificity rates for detection of regional nodal metastases ranged from 65 to 97% and 49 to 90%, respectively [35-39].

### 2.3. Endoscopic Ultrasonography (EUS)

In comparative studies, EUS generally provides a more accurate prediction of T stage than does CT [40-42], although newer CT techniques (such as three-dimensional multidetector-row CT) and magnetic resonance imaging may achieve similar results in terms of diagnostic accuracy in T staging [39,43,44]. In contrast, accuracy for nodal staging (65 to 90%) is only slightly greater with EUS as compared to CT [40,45-50]. EUS-guided fine needle aspiration of suspicious nodes and regional areas adds to the accuracy of nodal staging [51].

Most errors in staging with EUS are due to understaging of nodal involvement and the depth of primary tumor invasion; however, overstaging can also occur that is attributed to inflammation around the tumor or within the LNs [50]. EUS is not recommended for pretreatment evaluation of gastric cancer in the guidelines from the National Comprehensive Cancer Network (NCCN) [52].

### 2.4. Positron Emission Tomography (PET)

The role of PET using 18-fluorodeoxyglucose (FDG) in the preoperative staging of gastric adenocarcinoma is evolving. From the standpoint of locoregional staging, integrated PET/CT imaging can be useful to confirm malignant involvement of CT-detected lymphadenopathy [53]. However, this usually does not impact the decision to proceed to surgery. Furthermore, a negative PET scan is not helpful because even large tumors with a diameter of several centimeters can be falsely negative if the tumor cells have fairly low metabolic activity. Furthermore, most diffuse-type gastric cancers (signet ring carcinomas) are not FDG avid [54-58]. The main benefit of PET is that it is more sensitive than CT for the detection of distant metastases [42,58-60]. An important caveat is that the sensitivity of PET scanning for peritoneal carcinomatosis is only approximately 50% [61]. Thus, PET is not an adequate replacement for staging laparoscopy. NCCN guidelines for preoperative evaluation of gastric cancer suggest integrated PET/CT [52].

### 2.5. Sentinel Lymph Node (SLN) Biopsy

The application of the SLN technique in gastric cancer began in the late 1990s. Intraoperative subserosal or preoperative endoscopic submucosal injections can be used for the administration of blue dye or radioactive tracer. Identification of the SLN by means of a radiolabeled colloid and perioperative detection with a gamma probe has the disadvantage of radioactive tracing not only from LNs but also from the adjacent injection site. Most experience has therefore been gained with blue dye, but blue dye flows through and travels to the next LNs in line. The results reported in the literature on SLN biopsy in gastric cancer are widely divergent. Many authors from Asia reported an accuracy of more than 98% [62-64], in particular in early stages (T1-T2) [65], whereas other series from Western countries, the accuracy was about 80% [66-68], with the false negative SLN rate ranging from 15% to 20% [66-68]. The main reason for the poor accuracy could be the variability of the lymphatic routes in the gastric region, resulting in a high rate of skip metastases. Regarding the utility of SLN navigation in an attempt to detect the nodal basin, many issues are still to be resolved and further studies are recommended before this method can be introduced into daily practice.

## 3. LN Dissection

Complete surgical eradication of a gastric cancer with dissection of adjacent LNs represents the best chance for long-term survival. The choice of operative method for gastric cancer depends upon the location of the tumor in the stomach, the clinical stage, and the histological type. The major surgical considerations include the extent of luminal resection (total *versus* distal gastrectomy) and the extent of LN dissection.

### 3.1. EGC

Endoscopic resection is currently the standard treatment for EGC without the possibility of LN metastasis in Japan [69], as in the other countries, and is increasingly gaining acceptance as a therapy for EGC [70,71]. On the other hand, gastrectomy with LN dissection is required in cases of possible node metastasis [72] because the presence of LN metastasis has a strong adverse influence on patient prognosis [73,74]. In Japan, endoscopic submucosal dissection (ESD) is indicated for a differentiated mucosal cancer smaller than 2 cm in diameter [75] because risk for LN metastasis is negligible [17]. Recently, by using a large database involving more than 5000 patients who underwent gastrectomy with meticulous D2 level LN dissection, Gotoda and colleagues [17] were able to define the risk of LN metastasis. They revealed that submucosally invasive gastric cancer (similar to mucosal cancers) and tumor size larger than 3 cm with lymphatic or vessel involvement are significantly correlated with an increased risk of LN metastasis, and cancers penetrating deeply into the submucosal layer are most likely to be associated with LN metastasis. The extended indication including: (i) differentiated-type mucosal cancers without ulcerative findings, regardless of tumour size; (ii) differentiated-type mucosal cancers with ulceration findings, <30 mm; (iii) undifferentiated-type mucosal cancers without ulceration findings, <20 mm; and (iv) differentiated-type minute submucosal cancers (SM1) without ulceration findings, <30 mm was proposed by several reports [76,77].

From analysis of data from 118 patients with submucosal invasion, Yasuda *et al.* suggested that optimal LN dissection levels are as follows: (1) local resection (D0) for lesions of <1 cm; (2) limited LN dissection (D1) for 1- to 4-cm lesions, and (3) extended LN dissection (D2) for lesions >4 cm in diameter. When submucosal invasion of a tumor resected locally by ESD extends more than 300 mm, additional gastrectomy and LN dissection are necessary [78]. However, patients with submucosal invasion are not necessary to undergo D2 lymph node dissection in the Japanese guideline. Further study of the optimal extent of LN dissection for early gastric cancer is expected.

### 3.2. AGC

#### 3.2.1. Standard LN Dissection for AGC

One of the most controversial areas in the surgical management of gastric cancer is the optimal extent of LN dissection. Japanese surgeons routinely perform extended LN dissection, a practice that some suggest at least partially accounts for the better survival rates seen in Asia, as compared to Western series [79]. The term “extended lymphadenectomy” variably refers to either a D2 or D3 LN dissection. In present article, D3 was equivalent to D2+ which was described in the latest Japanese guideline in 2010 [80].

The draining LNs for the stomach can be divided into 16 stations: stations 1 to 6 are perigastric, and the remaining 10 are located adjacent to major vessels, behind the pancreas, and along the aorta.

- D1 l LN dissection refers to a limited dissection of only the perigastric lymph nodes.
- D2 LN dissection is an extended LN dissection, entailing removal of nodes along the hepatic, left gastric, celiac, and splenic arteries as well as those in the splenic hilum (stations 1-11).
- D3 dissection is a superextended LN dissection. The term has been used by some to describe a D2 lymphadenectomy plus the removal of nodes within the porta hepatis and periaortic regions (stations 1-16), whereas others use the term to denote a D2 LN dissection plus periaortic nodal dissection (PAND) alone [3]. Most Western surgeons (and the American Joint Committee on Cancer (AJCC)/International Union Against Cancer (UICC) TNM staging classification [81]) classify disease in these regions as distant metastases and do not routinely remove nodes in these areas during a potentially curative gastrectomy.

The arguments in favor of extended lymphadenectomy (D2 or D3 *versus* D1) are that removing a larger number of nodes more accurately stages disease extent and that failure to remove these nodes leaves behind disease in as many as one-third of patients, which would adversely affect survival [82-84]. A consequence of more accurate staging is minimization of stage migration [84,85]. The resulting improvement in stage-specific survival may explain, in part, the better results seen in Asian patients.

The influence of total LN count on stage-specific survival was studied in a series of 3814 patients undergoing gastrectomy for T1-3 N0-1 (classified according to the 1997 AJCC gastric cancer staging system and reported to the Surveillance, Epidemiology and End Results (SEER) database between 1973 and 2000) [86]. For every stage subgroup (T1/2N0, T1/2N1, T3N0, T3N1), survival was significantly better as more nodes were examined. Although cut-off point analysis revealed the greatest survival difference when 10 lymph nodes were examined, there were significant survival differences for cut-off points of up to 40 nodes examined, always in favor of a greater number of nodes in the specimen.

There are two main arguments against the routine use of extended LN dissection: the higher associated morbidity and mortality (particularly if splenectomy is performed to achieve extended LN dissection) and the lack of survival benefit for extended LN dissection in most large randomized trials.

### 3.3. Randomized Trials and Meta-analyses

Although many retrospective studies and only one randomized controlled trial (RCT) by a single institution in Taiwan suggest that extended LN dissection improves survival [87-89], multiple prospective randomized trials both in Asian and Western populations have failed to show a survival benefit with D2 *versus* D1 lymphadenectomy [90-92] or with D3 compared to D2 LN dissection [3,93-95]. The findings of the three largest trials are as follows.

#### 3.3.1. D1 *Versus* D2 Dissection

Medical Research Council (MRC) trial: The MRC trial randomly assigned 400 patients undergoing potentially curative resection to either a D1 or a D2 LN dissection [91]. Postoperative morbidity was significantly greater in the D2 group (46% *versus* 28%), as was operative mortality (13 *versus* 6%). Excess morbidity and mortality were clearly associated with the use of splenectomy and distal pancreatectomy to achieve complete node dissection. In a later follow-up, 5-year survival rates were no better for patients undergoing D2 compared to D1 dissection (33% *versus* 35%) [96].

Dutch trial: The largest randomized trial came from the Dutch Gastric Cancer Group and compared D1 with D2 LN dissection in 711 patients who were treated with curative intent [92,97]. This trial relied heavily upon input from a Japanese surgeon, who trained the Dutch surgeons in the technique of radical LN dissection and monitored the operative procedures. Despite these efforts to maintain quality control of the surgical procedures, both underremoval and overremoval of required nodal stations occurred, somewhat blurring the distinction between the groups. As was shown in the MRC trial, both postoperative morbidity (43% *versus* 25%) and mortality (10% *versus* 4%) were higher in the D2 group. Moreover, a statistically significant survival advantage in the radical dissection group was not observed, either in the initial report [92] or with longer follow-up [97,98], despite a significantly lower risk of recurrence. This was attributed to the detrimental impact of increased operative mortality in this group.

The conclusion of the Dutch trial was that D2 LN dissection could not be routinely recommended. However, many Asian surgeons consider that both the Dutch and the MRC trials are flawed. These studies are heavily criticized for poor quality control of the surgery and the postoperative care, unacceptably small hospital volume, high incidence of insufficient nodal dissection (noncompliance), and adoption of the more aggressive option of D2 dissection by routine use of pancreaticosplenectomy. The number of patients treated in an institute each year, termed hospital volume, showed clear negative correlation with hospital mortality. In the case of total gastrectomy, a certain incidence of morbidity is expected with this surgery, thus requiring the knowledge and experience of managing the associated complications [99]. In 2006, a RCT comparing D1 *versus* D2 (including D3 in the first edition of the Japanese Classification of Gastric Carcinoma) showed for the first time superiority of D2 over D1 dissection in clinical trials [100]. Five-year overall survival was 60% and 54% in the D2 and D1 groups, respectively (*P* = 0.041). This study is a single institutional study with three participating surgeons; thus, generalizability remains uncertain, especially in low-volume hospitals. However, with their experience, D2 dissection can be carried out with quite low hospital mortality (0%) and provides better survival than does D1 dissection. Thus, these issues are disputable. In 2010 the 15-year follow up of the Dutch trial [101] was reported that D2 lymphadenectomy is associated with lower locoregional recurrence and gastric-cancer-related death rates than D1 surgery, despite the fact that D2 lymphandectomy was also associated with significantly higher postoperative mortality, morbidity, and reoperation rates. Further studies to clarify the survival benefit of extended LN dissection are necessary.

#### 3.3.2. Para-aortic Lymph Node Dissection

Japan Clinical Oncology Group (JCOG) trial 9501: The multicenter JCOG study 9501 randomly assigned 523 patients to D2 *versus* D3 (D2 + PAND) dissection. The overall perioperative complication rate in the D3 group was significantly higher than that in the D2 group (28.1% *versus* 20.9%), although there were no differences in major complications (anastomotic leak, pancreatic fistula, abdominal abscess, pneumonia), and perioperative mortality was very low (0.8%) in both groups [93]. Five-year recurrence-free survival rate (approximately 63% in both groups) and overall survival rate (70% *versus* 69%) were no better after extended LN dissection [3].

One of the confounding issues with the JCOG trial is that in subgroup analysis, patients with node-negative disease fared significantly better with the more aggressive operation than with D2 LN dissection. Conversely, patients who were node-positive fared significantly better with a D2 LN dissection than with more aggressive surgery. The reasons for these counterintuitive results are unclear. Nevertheless, the high survival rate in both groups is notable in view of the fact that over 60% of both groups had positive nodes. These data underscore the marked differences in outcome between gastric cancers arising in Western and Asian populations. Data from the JCOG trial, as well as those from other groups [93,102], suggest that a D2 dissection can be performed safely with a perioperative mortality rate that is under 2%. A meta-analysis of the JCOG trial and two other smaller randomized trials of D2 *versus* D3 (with PAND) dissection [94,95] concluded that resection of the paraaortic nodes was inferior to a D2 dissection in terms of safety and was without any survival benefit [103]. Thus, paraaortic lymphadenectomy cannot be considered a routine practice for surgical treatment of gastric cancer.

#### 3.3.3. Splenectomy for Dissection of LNs at the Splenic Hiatus

There were two RCTs related to splenectomy for gastric cancer, the Chilean trial [104] and the Korean trial [105]. Both demonstrated no significant differences in postoperative mortality and 5-year survival. The Chilean trial, however, showed higher postoperative morbidity, and the Korean trial showed significant differences in the incidence of operative complications. Therefore, these results did not support the use of prophylactic splenectomy to remove macroscopically negative LNs near the spleen in patients undergoing total gastrectomy for proximal gastric cancer.

In Japan, a RCT to evaluate splenectomy for upper-third AGC is ongoing [106]. This trial includes the evaluation of long-term survival, postoperative morbidity, mortality, and quality of life. Registration of about 500 patients has been completed, and the results of this study are awaited.

## 4. The Role of LN Metastasis as a Prognostic Factor

### 4.1. Number and Location of LN Metastases

The presence of LN metastasis (pN) is one of the most significant prognostic factors in patients with gastric cancer. However the classification of LN metastasis in patients with gastric carcinoma is controversial. In 1981, the Japanese Research Society for Gastric Carcinoma first proposed a classification based on the anatomical location of positive nodes, which was reviewed by the Japanese Gastric Cancer Association in 1998. In 1997 and 2002, the UICC and the AJCC proposed a new classification for N categories that was based on the number of metastatic LNs (N stage) [107,108]. Now, the UICC/AJCC classification is used most widely for the staging of gastric cancer [107-109]. It can provide a more accurate estimation of prognosis than the classification based on anatomical lymphatic spread. Some authors have pointed out the superiority of UICC/AJCC classification on the grounds of simplicity, reliability, and stratification; they have also mentioned some of the problems associated with it, such as stage migration [110-114]. In 2010, the Japanese guideline has adopted the N categories based on numbers [80].

A new prognostic tool, for the ratio between metastatic LNs and the total number of LNs examined (N ratio), was proposed. This new classification reflects the degree of LN metastasis and reduces stage migration [84,85,115,116]. However, the significance of the N ratio has not been evaluated in patients with <15 examined LNs. Xu *et al.* evaluated the prognostic value of the N ratio staging system compared with the N stage classification when <15 LNs were examined in gastric cancer patients. N ratio categories were identified as follows: N ration 0, 0%; N ratio 1, 1% to 9%; N ration 2, 10% to 25%; N ration 3, >25%. They concluded that the positive N ratio is an independent prognostic factor, regardless of the number of LNs examined [117].

### 4.2. Micro-LN Metastasis

Micrometastasis was defined as the presence of tumor cells—single or in small clusters—detected only by cytokeratin specific immunostaining that could not be detected by ordinary H and E staining. There are specificities of several different antibodies, such AE1/AE3 (Boehringer Mannheim, Indianapolis, IN, USA), KL-1 (Immunotech, Marseilles, France), and CAM5.2 (Becton Dickinson, San Jose, CA, USA). Yasuda *et al.* demonstrated that LN micrometastasis is an independent prognostic indicator for patients with histologically node-negative gastric cancer invading the muscularis propria or deeper (T2 or T3) [4]. In addition to the presence of LN micrometastasis, the number and level of micrometastases in the LNs were strongly associated with the survival time of patients. It is controversial whether LN micrometastasis detected by immunohistochemistry predicts the clinical outcome of patients with histologically node-negative gastric cancer [5-9]. Nakajo *et al.* reported that LN micrometastasis correlated with a significantly worse survival rate in patients with T1 or T2 tumors [8]. Cai *et al.* also found a significant relation between LN micrometastasis and poor prognosis in patients with T3 gastric cancer [9]. However, Fukagawa *et al.* showed that the presence of LN micrometastasis did not affect survival in a large number of patients with T2 gastric cancer [6]. Nevertheless, although immunohistochemical detection of micrometastasis has not spread worldwide because of the complexity of the immunohistochemical technique used in Japan, this parameter may be helpful for deciding treatment strategies for adjuvant chemotherapy.

### 4.3. Extra-LN Metastasis

Extranodal metastasis, comprising cancer cells in soft tissue discontinuous with the primary lesion, is found during routine examination of about 10–28% of resected gastric carcinoma specimens [118]. According to the UICC, this type of tumor spread should be regarded as LN metastasis if the nodule has the form and smooth contour of a LN, but should otherwise be regarded as part of the primary tumor [107]. Some studies have, however, suggested that such tumor extension represents peritoneal seeding from either the primary tumor or metastatic LNs. Etoh *et al.* reported that extranodal metastasis was closely related to a poor prognosis [119]. Moreover, Nakamura *et al.* described that classification of patients into a capsule rupture group or no capsule rupture group, on the basis of the status of extranodal spread, was important [120]. These reports support the notion that extranodal metastasis should be included in the clinical classification of gastric cancer.

## 5. Molecular Biological Findings of LN Metastasis

Although the phenomenon of lymphatic spread of tumor has been well recognized for over a century, the mechanisms by which cancer cells enter into and proliferate within the lymphatic system remain unclear [121,122]. Lymphangiogenesis, the growth of new lymphatic vessels, is believed to underlie LN metastasis [123]. Although there is a large amount of data regarding angiogenesis, there are few reports on lymphangiogenesis, and the correlation between lymphatic vessel density and metastasis to LNs is controversial. A number of lymphatic-specific proteins, such as podoplanin, LYVE-1, and prox-1, have been identified [124-126]. VEGF-C and VEGF-D are ligands for VEGFR-3 (Flt-4), a tyrosine kinase receptor that is expressed predominantly in lymphatic endothelial cells [127]. Recent reports have shown that overexpression of VEGF-C or VEGF-D induces tumor lymphangiogenesis and promotes lymphatic metastasis in mouse tumor models [128-130]. Several studies have shown that expression of VEGF-C and VEGF-D by tumor cells correlates well with LN metastasis of gastric carcinoma [10-12]. These results indicate that quantitative analysis of lymphangiogenic markers in gastric cancer may be useful in predicting metastasis of gastric cancer to regional LNs.

## 6. Conclusions

There is no doubt that gastrectomy with regional LN dissection is the most useful modality for the treatment of AGC. In Japan and Korea, gastrectomy with D2 lymphadenectomy is the gold standard of treatment for this cancer. However, several studies have shown that more extended resection than D2 surgery has no impact on survival. To improve locoregional control of gastric cancer, the development of modalities for accurate preoperative determination of the status of LN metastasis and the establishment of multimodal treatment involving chemotherapy or radiotherapy in addition to surgery is expected. Additionally, basic research to clarify molecular biological mechanism of LN metastasis is necessary to obtain more favorable survival in patients with gastric cancer.
